# Gait kinematics at trot before and after repeated ridden exercise tests in young Friesian stallions during a fatiguing 10-week training program

**DOI:** 10.3389/fvets.2025.1456424

**Published:** 2025-02-10

**Authors:** Esther W. Siegers, Jeanne I. M. Parmentier, Marianne M. Sloet van Oldruitenborgh-Oosterbaan, Carolien C. B. M. Munsters, Filipe M. Serra Bragança

**Affiliations:** ^1^Department of Clinical Sciences, Faculty of Veterinary Medicine, Utrecht University, Yalelaan, Utrecht, Netherlands; ^2^Pervasive Systems Research Group, EEMCS, University of Twente, Drienerlolaan, Enschede, Netherlands; ^3^Equine Integration, Groenstraat Hoogeloon, Hoogeloon, Netherlands

**Keywords:** horse, training, workload, gait analysis, fatigue, welfare, Friesian horse, breeding selection

## Abstract

**Background:**

Appropriate training is essential for equine athletes to improve fitness and ensure welfare. Young Friesian stallions must complete a 10-week training program for acceptance as breeding stallions. Earlier, this training program was demonstrated to induce overtraining.

**Objectives:**

To evaluate how this training program affects stallions’ trot locomotion variables in relation to fatigue.

**Study design:**

Prospective analytical study.

**Methods:**

3 or 4 years-old (*n* = 16) Friesian stallions performed three ridden indoor standardized exercise tests (SETs) in week-1 (SET-I; *n* = 15), week-6 (SET-II; *n* = 11) and week-10 (SET-III; *n* = 4), measuring heart rate (bpm) and lactate concentration (LA, mmol/L). Before and after each SET, stallions’ locomotion was measured with seven inertial sensors (EquiMoves, 200 Hz) during in-hand trot on a straight line. Stride characteristics, limb angular changes, and upper body kinematics were calculated. The within-measurement coefficient of variation (CV) was calculated for all parameters. Linear mixed models were used to analyze gait variables related to SET, pre-or post-SET and a peak LA ≥4 mmol/L during SETs.

**Results:**

Horses showed individual responses in gait kinematics to moderate fatigue. The range of motion of the withers (ROM_withers_) increased post SET-II and SET-III compared to post SET-I. In horses reaching LA ≥ 4 mmol/L, CV increased post SETs for several stride characteristics and upper body asymmetry. Upper body vertical movement asymmetry was above the described reference ranges in 69% of the horses.

**Main limitations:**

Number of horses used and only four horses managed to complete the 10-week training program as breeding stallions.

**Conclusion:**

The young Friesian stallions showed individually different responses in absolute gait kinematics after exercise and during an intense training program. The increased ROM_withers_ and CV of stride characteristics after SETs suggest an acute effect of fatigue on the locomotion pattern. Further investigation is warranted for the pronounced upper body movement asymmetry related to published asymmetry reference values.

## 1 Introduction

In the last few years, societal attitudes toward horses have changed. Equestrians should minimize negative welfare effects and avoid unnecessary harm to the horse (1, 2). Equine stakeholders named training management as one of the five essential domains in managing of sport horse health and welfare (3). An appropriate training program can contribute to improving performance and is also valuable for the welfare of equine athletes (4–6). Physical exercise can lead to fatigue. The term fatigue, however abstract, has many different classifications (7, 8). Exercise-induced fatigue can be acute or chronic and can refer to a (neuro) muscular deficit (peripheral) or decline in mental function (central) (7–11). Muscle fatigue can be defined as “a decrease in maximal force or power production in response to contractile activity” (7, 10) or as a “reversible loss of muscle force during work over time” (9). On neuromuscular level, the nervous system fails to recruit muscle motor units adequately when being in a fatigued state. This mechanism leads to a decrease in proprioception, motor control and movement coordination (11) and thus increases the risk of injuries (8, 10–12). Chronic fatigue can lead to underperformance or overtraining (10, 12). In athletes, delaying the onset of fatigue is an essential feature of an effective training program (12, 13). However, it is difficult to quantify fatigue reliably, and early fatigue detection is challenging as currently there is no single conclusive marker of fatigue (11). Recovery is seen as the time to restore physiological and mental triggers from (intense) exercise. Appropriate recovery time reduces injury risk and improves the quality of the subsequent training (4, 10, 12).

Quantitative gait analysis systems have become more affordable and practical for daily use (14, 15). Kinematic gait analysis using inertial measurement units (IMU’s) is a reliable and practical manner to objectively measure and document horses’ gait in clinical or research settings (14, 16) with good repeatability and reproducibility (17–19), especially when sensors are placed both on the upper body and on the limbs (20). Gait symmetry measurements are an important feature of objective gait analysis techniques and are applicable to symmetrical gaits (e.g., walk, trot, and tölt) on a straight line or lunge, and can be used in lameness assessment of horses (14). Gait parameters can also be compared using repeated measurements of the same horses over time (14) and it has been shown that fatigue can influence movement patterns in horses (21–24). However, this influence is different when measuring on a treadmill or in field conditions (25). In field conditions, reduced stride frequency, as well as the protraction of forelimbs and retraction of hind limbs, was shown in fatigued horses (25, 26).

Yearly, the Royal Friesian Horse Studbook (KFPS) selects young Friesian stallions as breeding stallions. Part of this selection is a studbook approval test consisting of a 10-week training program. Horses are brought together at a training facility to assess athletic capability for dressage and driving and evaluate character and health (27).

This study aimed to evaluate gait parameters before and after repeated ridden standardized exercise tests (SET) in relation to exercise-induced fatigue and medium to long-term fatigue in young Friesian stallions during a 10-week training program. We hypothesized that exercise-induced fatigue would lead to altered gait kinematics and a higher step-by-step variability. Additionally, we hypothesized that medium to long-term fatigue would result in a less expressive gait as shown by reduced stride length and reduced range of motion of limbs and upper body kinematics.

## 2 Materials and methods

The Animal Ethics Committee of Utrecht University approved all research procedures (reference number 5204-1-04, approval date 24 June 2020). Written owners’ informed consent was also obtained. The physiology and training load data of this study has been published elsewhere (5). It was demonstrated that a state of chronic fatigue due to an intense training load, was induced. During the 10 weeks, horses did not get enough recovery time between training sessions and showed signs of overreaching or overtraining indicated by higher heart rates (HR) and plasma lactate concentrations (LA) during SETs at the end of the training program compared to the start of the training program (5).

### 2.1 Horses

Data were collected from privately owned young Friesian stallions. Horses (*n* = 16), age 3 (*n* = 13) or 4 (*n* = 3) years, were selected by the KFPS to participate to the 10-week stallion training program as part of the selection process for studbook approval. Horses were assessed by studbook judges in the months before the training program repeatedly on multiple selection days. Selection criteria consisted of exterior traits, gait characteristics, ridden performance and character as well as genetic information and health assessments such as sperm quality, left laryngeal hemiplegia and osteochondrosis dissecans (27). Horses selected by the studbook were included in the study after owner consent. Horses were kept in individual stables at the same training facility and were fed an individual diet consisting of roughage and concentrates during the entire study. Water was provided ad libitum. Horses had daily turnout in a sand paddock or a horse walker. Horses underwent dressage and driving training for 10 weeks. The training program was determined by the head trainer of the studbook (KFPS) and details are described in Siegers et al. (5). Horses were trained by 4 experienced riders and 2 experienced drivers. The stallion’s behavior, stable manners and occurrence of veterinary problems were documented.

### 2.2 Study design

During the 10-week training program, three submaximal ridden standardized exercise tests (SETs, Table 1) were performed in week 1 (SET-I), week 6 (SET-II) and week 10 (SET-III), measuring HR and LA. Gait analysis was performed before and after each SET. Gait analysis data was collected on a 40 m straight line on a hard surface (indoor) in trot (twice up and down per session) to collect a minimum of 10–20 strides per measurement session for analysis. One person (ES) handled the horses for all measurements. Studbook judges assessed the stallions in week 6 and week 10. Stallions could be eliminated from the stallion test by failing to obtain a positive evaluation by the judges, or as a result of an injury or other illness assessed by a veterinarian assigned by the studbook.

**Table 1:** Protocol of a submaximal ridden standardized exercise tests carried out in an indoor arena (20×60 m) for young Friesian horses, measuring heart rate and plasma lactate concentration.

Time (min)	Exercise	Measurement	Speed
SET	Indoor Arena under saddle		
00:00–01.00	Trot		
01:00–05:00	Walk		~ 2 m/s
05:00–07:00	Left trot		~ 3.5 m/s
07:00–09:00	Right trot		~ 3.5 m/s
09:00–10:00		LA	
10:00–12:00	Canter-1		~ 5.0 m/s
12:00–13:00		LA	
13:00–15:00	Canter-2		~ 5.0 m/s
15:00–16:00		LA	
16:00–26:00	Walk		Recovery ~2 m/s
26:00–27:00		LA	

Speeds (m/s) are estimates. SET, standardized exercise test; LA, plasma lactate concentration.

### 2.3 Equipment

**Figure 1:**
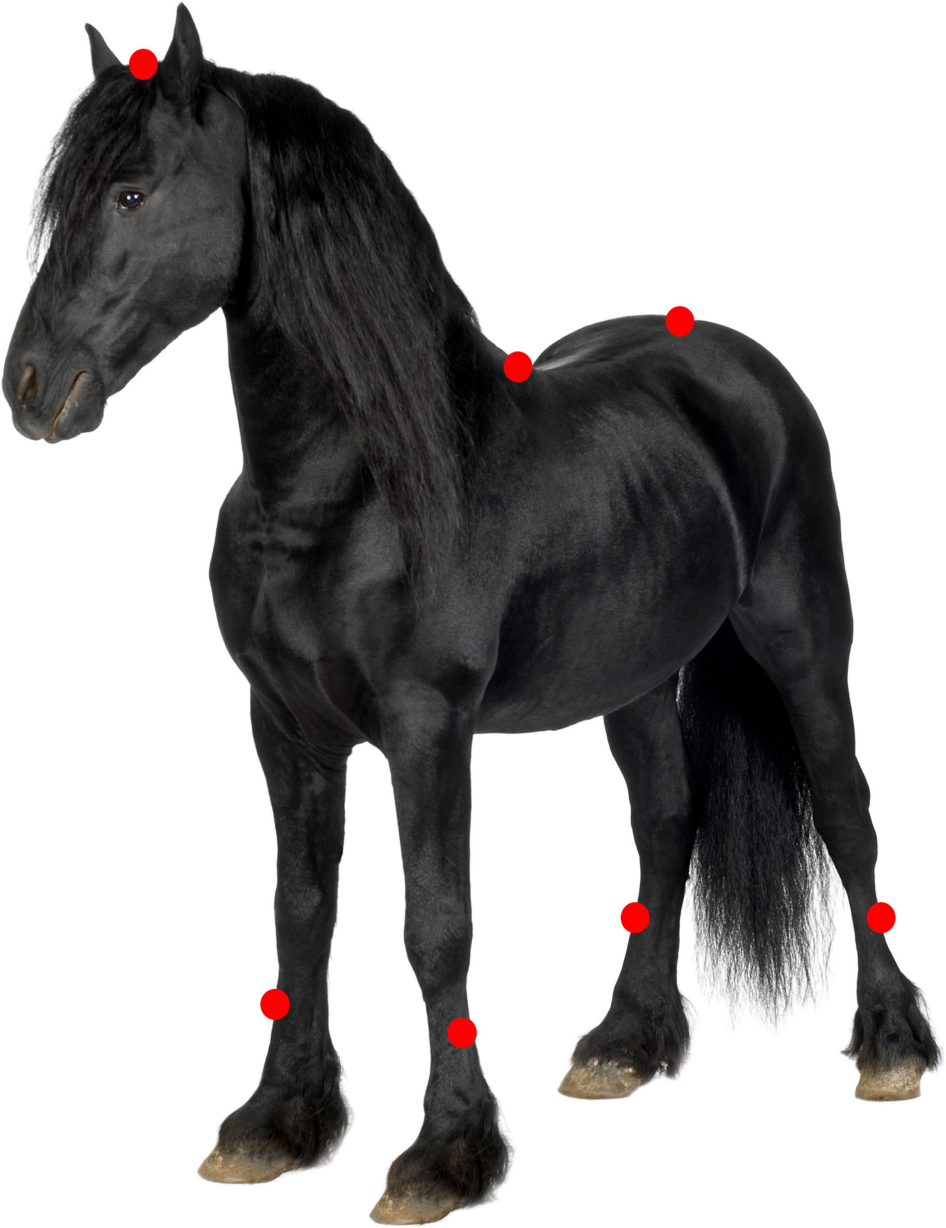
Locations of IMU sensor placements indicated on a Friesian horse. IMU placement sides are indicated with a red circle on the poll, withers, pelvis and on the mid-lateral aspect of the metacarpus/metatarsus on all 4 limbs.

During SETs, HR measurements were performed using a HR sensor (beats per minute (bpm), Polar V800 ® Polar Electro Oy, Kempele, Finland). Blood samples were collected by venepuncture of the left or right jugular vein using a sterile 2 mL syringe and a 23-gage needle to determine LA [mmol/L; Lactate Pro 2® (Arkray Inc. Kyoto, Japan)]. Ambient temperature (°C) and relative humidity (RH, %) were measured during the SETs using a heat stress wet bulb globe temperature (WBGT) device (Extech instruments HT30, Nashua, U.S.A.). Gait data collection was done using the wireless IMU-based EquiMoves software system (15). Seven IMU sensors (Inertia Technology B.V. Enschede, The Netherlands) with a sampling frequency of 200 Hz were placed as described by Bosch et al. on the following locations: the poll, the withers (using a girth), between the tubera sacrale of the pelvis and on the lateral aspect of the mid metacarpus/metatarsus of each limb. The sensors on the limbs were placed on a standard location (lateral aspect of the cannon bone) on dedicated brushing boots (Figure 1). Horses were accustomed to wearing brushing boots before the study. The IMU sensors were calibrated for 5 s according to the manufacturer’s recommendation.

### 2.4 Data collection

The SETs consisted of three incremental steps, HR was measured during the entire SET. Blood samples to determine LA were taken 30–60 s after each step, and after 10 min of recovery at walk (see Table 1). Gait analysis was performed in all horses within 2 h before each SET, and within 5 min after each SET.

### 2.5 Data processing

#### 2.5.1 Gait analysis data

Hoof events were automatically detected (28) and the signals were processed following the procedures described by Bosch et al. (15). The extracted parameters are described in Table 2. From the data collected from the IMU sensors, stride duration (sec), stance duration (sec), range of motion of the protraction of the distal limbs (ROM degrees), maximal protraction and retraction angles of the distal fore and hind limbs (degrees), and vertical displacement of the head (ROM_head_, mm), withers (ROM_withers_, mm), and pelvis (ROM_pelvis,_ mm) were calculated for each individual stride with custom MATLAB scripts (R2022b, MathWorks Inc., Natick, United States). Swing duration was calculated by the following equition:


swingduration=strideduration−stanceduration

**Table 2:** Detailed description of the calculated gait parameters used in this study.

Name	Abbreviation	Description
Stride duration (sec)		Time between hoof-on and subsequent hoof-on of the left hind limb
Stance duration (sec)		Time between hoof-on and hoof-off, mean of both fore/hind limbs
Fore	StanceF	
Hind	StanceH	
Swing duration (sec)		Stride duration minus stance duration, mean of both fore/hind limbs
Fore	SwingF	
Hind	SwingH	
Range of motion on the sagittal plane of the distal limbs (degree)		Range of motion of the sagittal limb angle as the angle range between maximal protraction and maximal retraction of both fore/hind limbs
Fore	ROM_F_	
Hind	ROM_H_	
Protraction distal limbs (degree)		Maximal forward protraction of the distal limb measured at the metacarpus/−tarsus in the sagittal plane
Fore		
Hind		
Retraction distal limbs (degree)		Maximal backward retraction of the distal limb measured at the metacarpus/−tarsus in the sagittal plane
Fore		
Hind		
Range of motion (mm)		Range of vertical displacement of the sensor
Head	ROM_head_	
Withers	ROM_withers_	
Pelvis	ROM_pelvis_	
Vertical displacement minimum difference (mm)		Measure of asymmetry, difference in minimum dorsoventral displacement of the sensor between left and right hoof-on/hoof-off in one stride
Head	HD_min_	
Withers	WD_min_	
Pelvis	PD_min_	
Vertical displacement maximum difference (mm)		Measure of asymmetry, difference in maximum dorsoventral displacement of the sensor between left and right hoof-on/hoof-off in one stride
Head	HD_max_	
Withers	WD_max_	
Pelvis	PD_max_	

From the gait analysis data, we created fore (F) and hind (H) parameters by averaging the individual left and right fore and hind limb values, respectively. Vertical movement asymmetry parameters were also obtained with the EquiMoves software, namely the differences in local minima and differences in local maxima reached by the head, withers and pelvis (HDmin/max, WDmin/max and PDmin/max respectively). In order to analyze changes in the amplitude of asymmetry only and not the side of the asymmetry, we used the absolute values of these parameters for the statistical analysis.

#### 2.5.2 Fatigue classification

Based on maximum LA values during the SET, horses were classified as more or less fatigued using LA concentration of ≥4 mmol/L as cut-off value. This value is generally considered as the anaerobic threshold (29, 30). Horses with a LA result ≥4 mmol/L were classified as more fatigued compared to horses with LA values <4 mmol/L. Horses completing the entire study were referred to as ‘*finishers*’.

#### 2.5.3 Heart rate data

Before processing, all HR data were visually checked for artifacts. If many artifacts were present (>5% of the measurement), data was not used for analysis. For HR analysis in the different gaits, the mean HR of the last 60 s in each gait and after 5 and 10 min of recovery were used.

### 2.6 Sample size estimates

Power calculations to determine the required number horses in the study were not possible, since this was a field study and the authors were restricted to use the number of horses included in the KFPS stallion selection process.

### 2.7 Statistical methods

#### 2.7.1 Outlier detection

All gait analysis data was checked for artifacts before analysis. All strides with a duration above or below 1.95 x standard deviation (s.d.) from the mean per individual horse per SET and per timepoint were considered outliers and were removed from the dataset.

#### 2.7.2 Data preparation

For the asymmetry parameters HD_min_, HD_max_, WD_min_, WD_max_, PD_min_ and PD_max_, absolute values were used for analysis to account for the amplitude of asymmetry rather than the side of the assymetry. The within measurement coefficient of variation (CV, %) was calculated for each parameter by the following equation:


$$CV=\left(\frac{s.d.}{\mathrm{mean}}\right)\times 100$$

### 2.8 Statistical analysis

Statistical analysis was performed using R-studio cloud 2024® (Boston, MA, United States), using the packages libraries dplyr (version 1.0.10), tidyr (version 1.2.0), ggplot2 (version 3.4.0), lme4 (version 1.1–35.1) and glmmTMB (version 1.1.5). Descriptive statistics are presented as mean ± s.d.

Linear mixed effect models were used for gait analysis and SET (HR, LA) data. Horse was used as a random effect. Whether horses finished the entire study (finishers) or not, SET number (I, II, III), pre/post SET and interaction effects between SET and pre/post SET measurement, and pre/post and LA ≥ 4 mmol/L were included as fixed factors in the linear mixed effect model. Deviations of normality and homogeneity of variances of the residuals were visually checked by examining the QQ plot and residual plot. Akaike’s Information Criterion (AIC) was used for model reduction. For important effects in the final model, 95% confidence intervals (95% CI) were calculated and presented as estimates and 95% CI.

Asymmetry results in our population compared with the reference values for Warmblood horses as described by Hardeman et al. (31) 2019 (12 mm for HD_min_, 9 mm for HD_max_, 3 mm for WD_min_ and WD_max_, 4 mm for PD_min_ and PD_max_) because they used the same gait analysis system as was used to validate the Equimoves® system and horses were also measured in-hand in trot on a hard surface.

To evaluate whether horses with more asymmetry before SET-I were related to elimination due to lameness or injuries, a logistic regression analysis was performed for elimination reason “lameness” (Y/N) using the factors HD_min_, HD_max_, WD_min_, WD_max_, PD_min_, and PD_max_. Odds ratios (OR) for relevant factors from the regression model were calculated.

## 3 Results

### 3.1 Descriptives

A total of 16 horses were included in the study. Twelve horses were withdrawn from the studbook stallion selection process prematurely: seven due to judge decisions and five due to injury/lameness. One stallion entered the training program 2 weeks after the start of the study because of an injury, and did not participate in SET-I. This horse completed the rest of the study. Resulting in 15 participating horses in SET-I. Of these horses, 10 participated in SET-II and the horse entering the program 2 weeks later was also participating in SET-II (total of 11 horses). From the horses participating in SET-II, four were also participating in SET-III. An overview of the number of horses participating to each SET, and available gait analysis and physiology data is presented in Table 3. A total of 1,332 strides were collected, of which 84 strides were outliers (6%) and were removed before data analysis. For each horse a mean of 20 ± 6.2 strides (min 4, max 34 strides) were available for analysis per sampling moment (SET-I/-II/III, before and after SET).

**Table 3:** Number of horses of which data is available for analysis per standardized exercise test in young Friesian stallions during a 10-week training program, and number of horses with a plasma lactate concentration ≥ 4 mmol/L during the standardized exercise test (percentage of participating horses).

	Total	Gait analysis		Physiology data	
		Before	After	HR and LA	LA ≥ 4 mmol/L
SET-I	15	15	15	15	5 (33%)
SET-II	11	11	9	8	5 (45%)
SET-III	4	3	4	4	4 (100%)

SET, standardized exercise test; LA, plasma lactate concentration (mmol/L); HR, heart rate (bpm).

### 3.2 Heart rate and plasma lactate concentration

Detailed descriptions of training data and heart rate and lactate results have been published elsewhere (5). HR and LA increased in SET-II and SET-III compared to SET-I, indicating that the training program led to a decreased fitness after 6 and 10 weeks (see Table 4).

**Table 4:** Mean ± s.d. results for heart rate and plasma lactate concentration in young Friesian horses during repeated ridden-submaximal standardized exercise tests after the first (Canter-1) and second (Canter-2) canter bouts.

	SET-I (*n* = 15)	SET-II (*n* = 8)	SET-III (*n* = 4)
HR			
Canter-1	141 ± 15	153 ± 17*	169 ± 7*
Canter-2	148 ± 17	164 ± 23*	175 ± 13*
LA			
Canter-1	3.1 ± 1.2	2.9 ± 1.0	5.4 ± 1.5*
Canter-2	3.2 ± 1.4	3.8 ± 1.7*	6.2 ± 1.6*

SET, standardized exercise test; LA, plasma lactate concentration (mmol/L); HR, heart rate (bpm). *Indicating a significant increase compared to SET-I, determined by statistical analysis using a linear mixed effect model and Akaike’s Information Criterion for model reduction.

#### 3.2.1 Gait analysis

Results of the linear mixed effect models of gait parameters are presented in Tables 5–8.

**Table 5:** Results of stride characteristics in young Friesian stallions before and after repeated submaximal ridden standardized exercise test in trot (in-hand), related to high (≥4 mmol/L) or low plasma lactate concentration.

		Main effects				Interaction effects		
Parameter	Intercept	SET-II	SET-III	After SET	Finishers	SET-II x after SET	SET-III x after SET	After SET x LA high
Stride duration (s)	0.77	−0.02*	0.00	−0.01	0.03*	0.01	−0.01	−0.02*
95% CI	0.75; 0.79	−0.03; −0.01	−0.01; 0.01	−0.02; 0.00	0.00; 0.07	−0.0; 0.02	−0.03; 0.01	−0.04; −0.01
% Change		−2.7	0.13	−1.1	4.4	1.2	−1.1	−3.1
Stance duration fore (s)	0.31	−0.01*	0.00	0.00	0.02*	−0.00	−0.02*	n.s.
95% CI	0.30; 0.32	−0.01; −0.00	−0.00; 0.01	−0.00; 0.01	0.01; 0.04	−0.01; 0.01	−0.03; −0.01	
% Change		−3.0	0.9	0.7	6.9	−0.09	−7.3	
Stance duration hind (s)	0.31	−0.01*	−0.02*	0.01*	n.s.	n.s.	n.s.	n.s.
95% CI	0.29; 0.33	−0.01; −0.01	−0.02; −0.01	0.00; 0.01				
% Change		−3.1	−4.8	2.0				
Swing duration fore (s)	0.46	−0.01*	0.00	−0.01	n.s.	n.s.	n.s.	−0.02*
95% CI	0.45; 0.48	−0.01; −0.00	−0.01; 0.01	−0.01; −0.00				−0.03; −0.00
% Change		−1.6	0.8	−1.6				−3.2
Swing duration hind (s)	0.48	−0.01*	0.02	−0.01	n.s.	0.01	−0.02*	−0.02*
95% CI	0.45; 0.50	−0.02; −0.00	0.01; 0.03	−0.02; −0.01		−0.00; 0.02	−0.04; −0.01	−0.03; −0.01
% Change		−2.2	4.5	−2.9		1.5	−4.3	−4.3

SET, standardized exercise test; LA, plasma lactate concentration (mmol/L). *Indicating important difference from SET-I/before SET or LA low. n.s. indicating that this factor was excluded from the statistical model. Number of horses: SET-I *n* = 15, SET-II *n* = 11, SET-III *n* = 4.

**Table 6:** Results of range of motion of head, withers and pelvis in trot (in-hand) in young Friesian stallions before and after repeated submaximal ridden standardized exercise test, related to high (≥4 mmol/L) or low plasma lactate concentration.

		Main effects				Interaction effects		
Parameter	Intercept	SET-II	SET-III	After SET	Finishers	SET-II x after SET	SET-III x after SET	After SET x LA high
ROM head (mm)	84.25	−5.40*	5.34	n.s.	11.99*	n.s.	n.s.	n.s.
95% CI	77.51; 91.15	−8.86; −1.94	−0.34; 11.03		2.58; 21.27			
% Change		−6.4	6.3		14.2			
ROM withers (mm)	121.20	−4.52	4.62	−6.6	n.s.	6.34*	6.41*	−6.59*
95% CI	115.63; 126.95	−8.82; −4.24	1.20; 8.04	−8.82; −4.24		3.43; 9.34	2.06; 10.76	−9.89; −3.20
% Change		−3.7	3.8	−5.4		5.3	5.3	−5.4
ROM pelvis (mm)	91.87	−1.50*	−7.23*	−1.34	9.18*	n.s.	n.s.	−3.46*
95% CI	85.44; 98.04	−2.85; −0.16	−9.53; −4.94	−3.01; 0.32	0.04; 18.34			−6.33; −0.59
% Change		−1.6	−7.9	−1.5	10.0			−3.8

SET, standardized exercise test; LA, plasma lactate concentration (mmol/L); ROM, range of motion. *Indicating important difference from SET-I/before SET or LA low. n.s. indicating that this factor was excluded from the statistical model. Number of horses: SET-I *n* = 15, SET-II *n* = 11, SET-III *n* = 4.

**Table 7:** Results of protraction and retraction angles of the limbs in trot (in-hand) in young Friesian stallions before and after repeated submaximal ridden standardized exercise test, related to high (≥4 mmol/L) or low plasma lactate concentration.

		Main effects				Interaction effects		
Parameter	Intercept	SET-II	SET-III	After SET	Finishers	SET-II x after SET	SET-III x after SET	After SET x LA high
ROM forelimbs (°)	95.63	1.33	3.55	−0.67	−6.50*	1.68*	−2.70*	−0.74
95% CI	93.63; 97.65	0.58; 2.08	2.35; 4.76	−1.48; 0.14	−9.44; −3.51	0.63; 2.73	−4.24; −1.16	−1.93; 0.44
% Change		1.4	3.7	−0.7	−6.8	1.8	−2.8	−0.8
ROM hind limbs (°)	61.57	−0.45	−2.17	−0.95	n.s.	1.32*	3.77*	n.s.
95% CI	60.26; 62.99	−1.08; 0.18	−3.15; −1.20	−1.59; −0.31		0.44; 2.21	2.60; 4.95	
% change		−0.7	−3.5	−1.5		2.2	6.2	
Hoof on protraction forelimbs (°)	25.43	0.22	−0.66*	0.27	−2.33*	n.s.	n.s.	n.s.
95% CI	24.63; 26.22	−0.13; 0.58	−1.22; −0.11	−0.045; 0.58	−3.51; −1.19			
% Change		0.9	−2.6	1.1	−9.2			
Hoof on protraction hind limbs (°)	26.49	−0.69	−2.92	1.13	n.s.	1.57*	1.63*	n.s.
95% CI	24.12; 28.85	−1.47; 0.9	−4.06; −1.79	0.34; 1.93		0.47; 2.66	0.17; 3.09	
% Change		−2.6	−11.0	4.3		5.9	6.2	
Hoof off retraction forelimbs (°)	−41.56	−0.85	−0.27	0.94	n.s.	−1.58*	−0.33	n.s.
95% CI	−43.14; −40.05	−1.32; −0.37	−1.02; 0.48	0.45; 1.42		−2.24; −0.90	−1.21; 0.56	
% Change		−2.0	−0.7	2.3		−3.8	−0.8	
Hoof off retraction hind limbs (°)	−23.60	−1.19*	−2.05*	−0.32	n.s.	n.s.	n.s.	1.96*
95% CI	−26.42; −21.14	−1.66; −0.73	−2.85; −1.26	−0.87; 0.23				0.67; 3.26
% Change		−5.1	−8.7	−1.3				8.3

SET, standardized exercise test; LA, plasma lactate concentration (mmol/L); ROM, range of motion. *Indicating important difference from SET-I/before SET or LA low. n.s. indicating that this factor was excluded from the statistical model. Number of horses: SET-I *n* = 15, SET-II *n* = 11, SET-III *n* = 4.

**Table 8:** Results of HD_min_, HD_max_, WD_min_, WD_max_, PD_min_ and PD_max_ in trot (in-hand) in young Friesian stallions before and after repeated submaximal ridden standardized exercise test, related to high (>4 mmol/L) or low plasma lactate concentration.

		Main effects				Interaction effects		
Parameter	Intercept	SET-II	SET-III	After SET	Finishers	SET-II x after SET	SET-III x after SET	After SET x LA high
HD_min_ (mm)	**20.98**	2.14	11.09*	n.s.	n.s.	n.s.	n.s.	n.s.
95% CI	13.48; 28.40	−1.21; 5.49	5.90; 16.28					
% Change		10.2	52.9					
HD_max_ (mm)	**17.43**	1.04	7.95*	−4.03	4.31	7.526*	1.58	5.70*
95% CI	12.63; 22.49	−2.95; 5.04	1.49; 14.45	−8.39; 0.33	−1.69; 10.13	1.89; 13.16	−6.73; 9.90	0.064; 11.03
% change		6.0	45.6	−23.1	24.7	43.2	9.1	32.7
WD_min_ (mm)	**5.37**	3.42	1.61	0.93	n.s.	−1.82*	−1.23	0.3
95% CI	3.41; 7.25	2.17; 4.66	−0.38; 3.60	−0.43; 2.27		−3.57; −0.08	−3.79; 1.33	−1.56; 2.18
% Change		63.6	30.0	17.1		−33.9	−22.9	5.5
WD_max_ (mm)	**11.68**	2.18*	1.087	−0.11	n.s.	n.s.	n.s.	−2.45*
95% CI	8.79; 15.12	1.06; 3.30	−0.81; 2.99	−1.47; 1.25				−5.52; −0.12
% Change		18.6	9.3	−0.9				−20.9
PD_min_ (mm)	**9.14**	−1.78*	0.57	−0.60	n.s.	n.s.	n.s.	0.67*
95% CI	6.51; 11.64	−2.86; −0.70	−1.27; 2.41	−1.94; 0.74				1.26; 5.77
% Change		−19.5	6.2	−6.6				7.4
PD_max_(mm)	**8.76**	3.79*	0.20	−0.0024	2.12	n.s.	n.s.	−2.20
95% CI	6.37; 11.09	2.63; 4.87	−1.67; 2.09	−1.39; 1.39	−0.94; 5.13			−4.47;0.046
% Change		42.8	2.3	−0.03	24.2			−25.2

SET, standardized exercise test; LA, plasma lactate concentration (mmol/L); HD_min_, head minimal vertical displacement difference; HD_max_, head maximal vertical displacement difference; WD_min_, withers minimal vertical displacement difference; WD_max_, withers maximal vertical displacement difference; PD_min_, pelvis minimal vertical displacement difference; PD_max_, pelvis maximal vertical displacement difference. *Indicating important difference from SET-I/before SET or LA low. n.s. indicating that this factor was excluded from the statistical model. Number of horses: SET-I *n* = 15, SET-II *n* = 11, SET-III *n* = 4.

For all gait parameters, horses showed different individual responses to the training program and the effect of fatigue (after SET and LA ≥ 4 mmol/L). As examples, the individual responses for ROM_withers_, forelimb forward protraction and StanceH are shown for each horse in Supplementary Figure S1.

#### 3.2.2 Coefficient of variation

**Figure 2:**
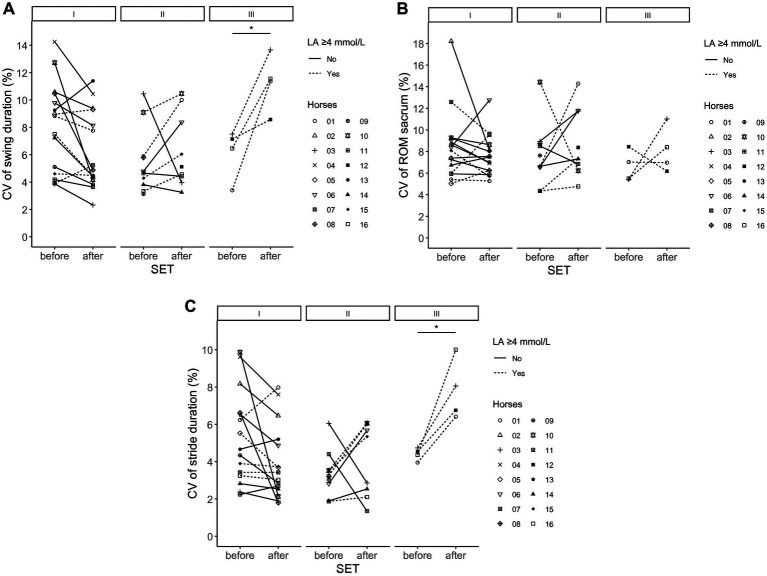
**(A–C)** results of mean coefficient of variation of swing duration of the hind limbs **(A)**, ROM of the plevis/sacrum **(B)** and stride duration **(C)** in trot (in-hand) in young Friesian stallions before and after repeated submaximal ridden standardized exercise test during a 10-week training program related to high (≥4 mmol/L, dotted line) or low (<4 mmol/L, solid line) plasma lactate concentration. Each marker represents the mean results per individual horse.* indicating important difference from before SET. SET, standardized exercise test; ROM, range of motion; CV, coefficient of variation (%).

For stride duration parameters there were important increases in CV in more fatigued horses (Figure 2). The variation in stride duration (intercept 6.2; 95% CI 4.6, 7.7) and StanceH (intercept 7.8; 95% CI 6.1, 9.3) was higher in more fatigued horses shown by higher CV after SETs in horses with LA ≥ 4 mmol/L (2.5, 95% CI 0.8, 4.1 and 4.3; 95% CI 2.4, 6.1 respectively) compared to before SET. Variation in SwingH (intercept 7.3; 95% CI 5.6, 8.9) increased after SET-III compared to before SET-III (7.6; 95% CI 3.9, 11.2).

The CV of the asymmetry parameters HD_max_, WD_min_ and PD_max_ also changed in more fatigued conditions, but were not different for the other asymmetry parameters. The CV of WD_min_ (intercept 5.4; 95% CI 3.4, 7.3) increased after SET-III (29.2; 95% CI 4.6, 53.8) compared to before SET-III, but was overall lower after the SETs in horses with LA ≥ 4 mmol/L (−15.7; 95% CI −28.2, −3.2). The CV of HD_max_ (intercept 119.0; 95% CI 97.0, 141.1) was lower in SET-III (−34.1; 95% CI 65.3, 2.9) compared to SET-I. The CV of PD_max_ (estimate 64.3; 95% CI 51.0, 77.7) increased after SET-II (23.2; 95% CI 51.0, 77.7) compared to before SET-II, but was overall lower in SET-III compared to SET-I.

The CV of the ROM_head_, ROM_withers_, ROM_pelvis_, ROM_protF_ and ROM_protH_ did not change significantly.

#### 3.2.3 Asymmetry parameters

The asymmetry parameters HD_min_, HD_max_, WD_min_, WD_max_, PD_min_ and PD_max_ were measured twice per SET per horse, resulting in a total of 342 individual average data points. The mean ± s.d. and percentiles per parameter and per SET are presented in Supplementary Table S1 and are compared to reference ranges for Warmblood horses (31). Eleven out of 16 stallions (69%) had no upper body asymmetry measurements within the reference range, and all horses had at least one measurement above the reference range on all occasions (SET-I, -II and III). From the measurements exceeding the reference value (*n* = 334, 97.6%), only eight were less than 10% above the reference value. A total of 259 out of 334 (75.7%) measurements were more than 50% above reference value. One horse had a PD_min_ of 30.7 mm before SET-II (7.7 times higher than the reference limits). Both before and after SET-I, three measurements for HD_min_ were below the reference range but only two stallions had a HD_min_ within the reference range on both occasions.

In the logistic regression model only WD_min_ was related to elimination from the study due to an injury or lameness. The log Odds ratio was 0.093 (95% CI 0.046; 0.14), the OR was 1.097 (*p* < 0.001).

## 4 Discussion

In this study, it was demonstrated that individual young Friesian horses have different changes in limb and upper body kinematics in response to a submaximal exercise test and to a longitudinal fatiguing training program. In more fatigued conditions, horses showed a higher coefficient of variation in stride characteristics and asymmetry parameters. However, variation in ROM of limbs and upper body remained constant. Additionally, 97.6% of the upper body asymmetry parameters measured were above the described reference values for owner-sound Warmblood horses measured in-hand in trot on a straight line (31), with 69% of the participating young Friesian horses having all parameters on all SETs above the reference range.

Gait parameters acquired by objective gait analysis systems can be compared using repeated measurements of the same horses over time (14). In the present study, young Friesian horses were measured twice on 1 day (before and after SET), and on three different days (SET-I, II and III) over time. Hardeman et al. (31, 32) showed that between-horse measurements have substantially larger variation than within-horse measurements on different measurement moments, demonstrating the consistency in the locomotion pattern of individual horses. This enables repeated gait evaluation to compare the horse with itself. The larger variation in head parameters compared to withers and pelvis measurements has been shown previously (23, 31, 33). Excitement and environmental stimuli, as well as the handler of the horse can cause larger and perhaps more asymmetric head movements in horses, especially in our young stallion population. In this study, we tried to reduce these effects as much as possible by trotting the horses in a quiet surrounding where the horses were accustomed to and by using the same handler for all horses. However, some excitement could not be prevented, and results for ROM_head_ and asymmetry of the head should be interpreted cautiously.

The Friesian horses participating in the present study were pre-selected by the studbook to become an approved studbook/breeding stallion. Gait quality is one of the features in the selection process of these stallions (27), but is a subjective parameter and consists of elements such as ground cover and self-carriage which are judged by studbook judges. Stride length is seen as the gold standard to quantify ground coverage and is explained by kinematic measures of the limbs such as stance duration, swing duration and forelimb retraction and hind limb protraction angles (34–36). A larger stride length in Warmblood horses in trot is scored positively by judges (34, 37, 38). Friesian stallions completing the entire study had slightly longer strides durations (4,4%), ROM_head_ and ROM_pelvis_ compared to non-finishers. This can be explained by more expressive gaits resulting in more positive judge scores for these horses, and thus longer stay in the studbook approval program. Remarkably, over all measurements, the protraction angle of forelimbs and ROM_protF_ were lower in finishers. Thus the longer stride duration in finishers was not obtained by more protraction of the forelimbs. No other stride duration parameter was significantly different in finishing horses.

To the authors knowledge, no studies have been published yet on gait analysis in Friesian horses compared to other breeds. However, subjectively, Friesian horses have a different movement pattern compared to Warmblood or Thoroughbred horses. Rhodin et al. (39) described gait parameters of a group of 19 sound adult Warmblood riding horses and 23 healthy adult Iberian horses. Hardeman et al. (32) described gait parameters in 12 healthy adult Warmblood performance horses. When comparing stride kinematics of the Friesian stallions in the present study with the results of Hardeman et al. (32) and Rhodin et al. (39), mean stride duration (0.77 s) is comparable to reported mean of Warmblood horses (0.78 and 0.76 s), but higher than in Iberian horses (0.74 s). The expressive gait of the selected Friesian stallions seems to be represented by a longer swing duration (0.46 s fore, 0.48 hind) compared to Warmbloods (0.39 and 0.30 s fore and 0.46 s hind) and Iberian horses (0.30 s fore and hind) (39, 40). The ROM of the upper body kinematics of the young stallions were higher than published results of other breeds using the same IMU sensors. The estimate of ROM_head_ of the young Friesian horses was 80.73 mm (95% CI 74.98; 86.40), compared to a median of 67.21 mm (95% CI 51.99; 87.75) (32) and estimated marginal mean of 78.1 mm (95% CI 70.5–85.7) in mature Warmblood horses, and 63.7 mm (95% CI 56.8; 70.6) in mature Iberian horses (39). The ROM_withers_ in the Friesian horses was even more different from Warmbloods and Iberian horses. The ROM_withers_ was 29 mm and 35 mm larger in the young Friesian stallions than in Warmbloods and 55 mm larger than in Iberian horses. The ROM_pelvis_ was 3 mm and 2 mm larger in the Friesian stallions than in Warmblood horses and 28 mm larger than in Iberian horses (32, 39). The Friesian horses used in this study are an elite group as being in the selection of becoming an approved breeding stallion, and thus may not be fully representable for the entire breed. Taking that into account, the results do indicate different movements compared to adult Warmblood and Iberian horses. In the present study, ROM_head_ and ROM_pelvis_ were higher in finishers. The higher upper-body vertical ROM might be related to more expressive and higher vertical impulsion movement of the horse, and thus higher judge scores.

A ridden submaximal exercise test was used in the present study. The workload of this test is highly representative for the normal exercise for the young Friesian stallions and induced a relevant level of fatigue as measured by lactate concentrations exceeding the anaerobic threshold of 4 mmol/L during the SET in many of the participating horses (29, 30). Typically, cardiorespiratory fitness improves after several weeks of training (6, 41–43). However, HR and LA response to the SET in the present study increased markedly, implying a fatiguing training program. The workload during SETs was standardized, and the negative physiologic response suggests overreaching/overtraining of these young horses (44–46). In people, the effects of fatigue on gait parameters varies among individual athletes and is influenced by training status, running technique and field or treadmill exercise (47, 48). Back et al. (49) demonstrated individual different changes in gait kinematics in unexperienced 2.5 year-old Warmblood horses after a 10-week training protocol. Similarly, in the present study, no consistent changes in gait kinematics were found. Horses showed individually different responses in gait kinematics after SETs and in repeated SETs during a fatiguing training period. For example stride duration increased in six and decreased in nine out of 15 horses after SET-I compared to before SET-I. This implies that gait response to moderate fatigue does not follow a single pattern in young Friesian horses.

Speed could not be measured during gait analysis or SETs because the tests were performed indoors where satellite (GPS) signal was poor. In order to keep speed as constant as possible, one handler trotted all horses. Additionally, riders were asked to keep their speed as constant as possible during the SETs. In the present study, in SET-II stride duration, StanceF, StanceH, SwingF and SwingH were all lower compared to SET-I. It can therefore be suggested that speed during gait data collection in SET-II was lower compared to SET-I and SET-III. However, according to Hardeman et al. (31, 32) differences in trotting speed do not affect upper body ROM and asymmetry outcomes. Therefore, the ROM results of SET-II can be considered as valid.

The effect of acute fatigue was studied by gait analysis before and after SETs but is also influenced by medium to long term fatigue due to the fatiguing training program. Horses with LA ≥ 4 mmol/L reduced their stride duration after SETs by reducing swing duration, but maintaining stance duration constant. After SET-III, where all horses had LA ≥ 4 mmol/L, StanceF and SwingH were reduced. Darbandi et al. (23) collected gait analysis data before and after a SET from 60 horses to develop a model to detect fatigue using machine learning techniques. The 16 Friesian stallions from the current study were part of this dataset. In those 60 horses, swing duration decreased after SETs as well and was related to fatigue (23). In field conditions, Takahashi et al. (26) showed decreased speed, stride frequency and stride length in Thoroughbreds comparing the first and the second lap in a race using a stationary high-speed camera system. Horses did not lengthen their body as much as they did at the start of the race (26). It has been shown that muscle fatigue can lead to a decreased power output (9) and thus result in a shorter swing phase in horses. There are several studies in sport horses, using IMU’s, evaluating the effect of fatigue on stride duration, but studies used all different methodologies leading to conflicting results among studies and making comparisons difficult (24, 50, 51). In the present study, ROM_withers_ increased after SET-II and SET-III, but ROM_withers_ and ROM_pelvis_ was lower after SETs in horses with LA ≥ 4 mmol/L. A lower SwingH could be the result of less push from the hind limbs due to muscle fatigue and can be directly associated with less motion of the withers and pelvis, but when stride length characteristics remained constant, dorsoventral motion of the withers was increased after SETs. Studies in human and equine athletes also show increased movement of the trunk: in human runners vertical lift increases in fatigued individuals (47), especially in less experienced runners (52). Additionally, peak forward trunk lean increases (48) leading to a less economical running technique. In a small study by Colborne et al. (53), dorsoventral displacement was larger under fatigued conditions than under non-fatigue conditions in Thoroughbreds on a treadmill. However, this was a small pilot study with vertical wither distance results for only 2 horses (53). A possible explanation of the increased vertical motion of the withers of the Friesian stallions in acute fatigue could be a diminished trunk stability leading to more movement of the withers.

In the parameters ROM_F,_ ROM_H_, protraction and retraction angles of fore and hind limbs and in asymmetry parameters, no consistent effect of fatigue or training was found. This is in contrast to the findings of other studies (23, 25, 26, 53), where ROM_protF_, ROM_protH_, and hind limb protraction/retraction angles decreased in more fatigued horses. However, three of these studies cannot be directly compared to the young Friesian stallions, since they used galloping racehorses, either on the treadmill or during a race (25, 26, 53). It is possible that horses in in-hand trot after ridden exercise respond differently to fatigue compared to racehorses measured in gallop during the fatiguing exercise.

Beside changes in absolute measurements, stride by stride variation can be related to fatigue due to adaptations of the neuromotor system (54). In a fatigued state, the nervous system lacks ability to recruit motor units adequately. This leads to a lower muscular power output, but also increased variability in movement and altered movement coordination (11). Hardeman et al. (31, 32) showed that variation reduced in repeated gait analysis measurements in horses, and individual variation was highest on the first measurement day compared to a second and third measurement day in healthy horses. In the present study, the coefficient of variation was not significantly higher during measurements before SET-I compared to SET-II and SET-III. However, the CV in stride duration increased by 54% after SETs in horses with LA ≥ 4 mmol/L, showing that more fatigued horses have less consistent stride durations. Upper body kinematics and limb ROM did not show a change in variation in the study, but asymmetry parameters did show more variation in more fatigued conditions. Similarly, in human athletes it has been shown that for some parameters, such as knee kinematics, variability increases, while in other parameters, for example ground reaction force, variability decreases in fatigued individuals (54, 55).

The horses in the present study showed marked upper-body asymmetries during all measurements. Reference values have been reported to evaluate what can be considered as ‘normal’; however these are population and measurement system-specific and there is overlap between “owner sound” horses and horses with induced lameness (16, 33). Eleven out of 16 young Friesian (69%) stallions in the present study had no measurements within the reference values as determined for adult Warmblood horses by Hardeman et al. (31), using the same sensor system. None of the horses had results within this reference range for HD_max_, WD_max_, PD_min_ and PD_max_ before SETs. After SETs, none of the young Friesian horses had results within reference range for the same parameters and WD_min_. The majority of these measurements exceeded the reference range for between day variation by more than 10% (326 out of 342 measurements) or 50% (259 out of 342 measurements) and had values associated with lameness that is visible for the human eye. This can be a potentially concerning result in a group of young and presumably healthy horses. All horses were evaluated by an experienced veterinarian before entering the study, and all horses were judged as “fit to compete.” Also, horses were excluded from the study if lameness was evident as determined by trainer, rider, judge or veterinarian. Other studies in presumably healthy horses report similar high number of horses with motion asymmetries of up to 70% (16, 31, 50, 56–58). It is questionable at what threshold asymmetry becomes clinically relevant. It is unlikely that all these horses are lame due to a painful (orthopedic) condition, therefore better understanding of the relationship between pain and measured asymmetries is needed. Pfau et al. (33) suggested higher reference values for asymmetry of the head (14.5 mm) and pelvis (7.5 mm) in Thoroughbred horses in trot in-hand on a straight line. Most measurements in the young Friesian stallions still exceed these limits, however the trot of a Friesian might not be comparable to a Thoroughbred. The horses in the present study were young, have had limited training before the study and have high upper body ROM compared to other breeds. The large upper body ROM demonstrated in our study compared to other breeds might lead to higher absolute asymmetry values, underlining the importance of breed-specific reference and possibly even age-or training level-specific values for asymmetry and lameness.

In the Friesian stallions, elimination from the study was related to WD_min_, but not to asymmetry parameters of head and pelvis. In the statistical analysis, horses with forelimb and hind limb asymmetries were combined in one group, therefore it was logical that only a relation with withers asymmetry was seen in the statistical model. We hypothesize that that more symmetric horses might have received better scores from the judges than the horses with a more asymmetric gait.

Limitations of the study include the small number of horses involved, mainly due to the selection process for Friesian stallions. Only four horses remained in the study until SET-III and became an approved studbook stallion for the Friesian Studbook. The factor that only a few horses completed the 10-week training program was statistically accommodated for with the use of the mixed effects model. Gait assessment was not conducted during the submaximal exercise tests (SETs) but before and after the SETs, and horses had a 10-min cool-down before the post-SET gait analysis, potentially affecting results. More uniform results might be obtained in the Friesian horses when gait kinematic data was collected at the peak of their fatigue. However, this would mean that horses should be measured during the ridden exercise tests and riders might influence the gait kinematics as well leading to factor contributing to the variation in the data. More intense saddle-based or treadmill tests could yield clearer results in future studies regarding investigating the effect of fatigue on locomotion parameters.

Overall, the findings in this study support the first hypothesis that stated that the fatigued young Friesian stallions would increase their step-by-step variability and gait kinematics. However, the second hypothesis, that stated that the young stallions would reduce stride length, ROM of limbs and upper body kinematics as a result of medium to long-term fatigue, could not be confirmed.

## 5 Conclusion

The young Friesian stallions showed individual changes in gait parameters after moderate intense exercise and during a fatiguing training program. An increased range of motion of the withers after exercise was a consistent finding. In more fatigued conditions, horses showed more variation in stride characteristics and asymmetry parameters. However, variation in ROM of limbs and upper body remained constant. Additionally, 69% of the young Friesian stallions showed higher asymmetry values in in-hand trot on a straight line compared to the published reference values for Warmblood sport horses. These high number of asymmetric horses have been described in other breeds and groups of presumably healthy horses. Thus, there is a necessity of breed-and possibly age-specific asymmetry reference values, and further evaluation of factors contributing to upper body asymmetries in healthy horses.

## Data Availability

The raw data supporting the conclusions of this article can be requested from the corresponding author.
