# Supplementary material for: Gait kinematics at trot before and after repeated ridden exercise tests in young Friesian stallions during a fatiguing 10-week training program
Source: Front Vet Sci. 2025 Feb 10;12:1456424. doi: 10.3389/fvets.2025.1456424 (PMC11848856; doi:10.3389/fvets.2025.1456424)
Supplement: Supplementary file 1 [file Table_1.docx]

Table S1: Mean ± s.d. and 5^th^, 25^th^, 50^th^, 75^th^ and 95^th^ percentiles of the absolute asymmetry parameter results per standardized exercise test compared to Warmblood reference ranges and number of horses with results below the reference range (out of total number participating horses for that SET) in trot (in-hand) in young Friesian stallions before and after repeated submaximal ridden standardized exercise test.

Abbreviations: SET, standardized exercise test; HD_min_, head minimal vertical displacement difference; HD_max_, head maximal vertical displacement difference; WD_min_, withers minimal vertical displacement difference; WD_max_, withers maximal vertical displacement difference; PD_min_, pelvis minimal vertical displacement difference; PD_max_, pelvis maximal vertical displacement difference

|  |  | Reference (mm) | Mean ± s.d.  (mm) | Number of horses < reference (total participating horses) | Percentiles | | | | |
| --- | --- | --- | --- | --- | --- | --- | --- | --- | --- |
| **Before SET** | | |  |  | 5^th^ | 25^th^ | 50^th^ | 75^th^ | 95^th^ |
| SET-I | HD_min_ | 12 | 20.6 ± 19.2 | 3 (15) | 1.62 | 7.35 | 15.80 | 27.72 | 62.18 |
|  | HD_max_ | 9 | 16.9 ± 14.0 | 0 (15) | 1.78 | 5.98 | 13.58 | 23.64 | 41.62 |
|  | WD_min_ | 3 | 8.2 ± 5.8 | 1 (15) | 0.60 | 3.59 | 7.46 | 11.56 | 19.29 |
|  | WD_max_ | 3 | 8.3 ± 6.8 | 0 (15) | 0.69 | 3.26 | 7.40 | 11.29 | 19.83 |
|  | PD_min_ | 4 | 10.2 ± 8.0 | 0 (15) | 0.72 | 3.83 | 8.47 | 14.72 | 25.99 |
|  | PD_max_ | 4 | 8.2 ± 6.6 | 0 (15) | 0.59 | 3.05 | 6.86 | 11.18 | 20.19 |
| SET-II | HD_min_ | 12 | 22.8 ± 19.4 | 0 (11) | 2.34 | 10.05 | 21.56 | 34.84 | 58.06 |
|  | HD_max_ | 9 | 18.3 ± 15.5 | 0 (11) | 1.38 | 7.32 | 15.46 | 23.45 | 55.02 |
|  | WD_min_ | 3 | 9.4 ± 7.4 | 0 (11) | 0.80 | 4.05 | 8.29 | 14.21 | 21.08 |
|  | WD_max_ | 3 | 10.6 ± 8.6 | 0 (11) | 0.78 | 3.91 | 7.92 | 14.94 | 25.54 |
|  | PD_min_ | 4 | 8.1 ± 6.8 | 0 (11) | 0.57 | 2.68 | 6.78 | 11.12 | 21.86 |
|  | PD_max_ | 4 | 12.5 ± 9.0 | 0 (11) | 1.29 | 5.65 | 10.48 | 17.19 | 31.12 |
| SET-III | HD_min_ | 12 | 36.4 ± 34.9 | 0 (3) | 2.35 | 12.26 | 27.67 | 46.76 | 109.88 |
|  | HD_max_ | 9 | 27.5 ± 23.4 | 0 (3) | 3.05 | 11.22 | 20.64 | 36.56 | 76.06 |
|  | WD_min_ | 3 | 8.7 ± 7.5 | 0 (3) | 0.32 | 2.17 | 5.46 | 13.51 | 21.78 |
|  | WD_max_ | 3 | 8.5 ± 6.6 | 0 (3) | 0.39 | 3.61 | 7.17 | 11.80 | 22.25 |
|  | PD_min_ | 4 | 11.5 ± 10.4 | 0 (3) | 0.84 | 2.94 | 11.09 | 14.13 | 22.11 |
|  | PD_max_ | 4 | 10.4 ± 6.4 | 0 (3) | 1.24 | 6.11 | 10.84 | 14.60 | 21.92 |
| **After SET** | | | | | | | | | |
| SET-I | HD_min_ | 12 | 18.3 ± 17.4 | 3 (15) | 1.69 | 7.01 | 13.64 | 24.19 | 58.10 |
|  | HD_max_ | 9 | 15.3 ± 12.8 | 0 (15) | 1.48 | 5.97 | 12.41 | 22.00 | 39.99 |
|  | WD_min_ | 3 | 8.3 ± 6.9 | 0 (15) | 0.74 | 2.96 | 6.31 | 11.64 | 22.71 |
|  | WD_max_ | 3 | 8.5 ± 6.6 | 0 (15) | 0.57 | 3.24 | 6.52 | 12.03 | 21.13 |
|  | PD_min_ | 4 | 11.0 ± 9.2 | 0 (15) | 0.91 | 3.48 | 7.66 | 16.71 | 28.23 |
|  | PD_max_ | 4 | 8.6 ± 6.4 | 0 (15) | 0.60 | 3.16 | 7.25 | 12.26 | 20.59 |
| SET-II | HD_min_ | 12 | 23.3 ± 22.7 | 1 (9) | 1.79 | 7.17 | 16.21 | 34.31 | 76.11 |
|  | HD_max_ | 9 | 25.9 ± 26.4 | 0 (9) | 1.89 | 8.47 | 17.29 | 36.27 | 83.09 |
|  | WD_min_ | 3 | 8.0 ± 5.8 | 0 (9) | 0.67 | 3.66 | 6.26 | 10.51 | 17.88 |
|  | WD_max_ | 3 | 10.2 ± 8.9 | 0 (9) | 0.49 | 2.77 | 6.70 | 13.18 | 25.95 |
|  | PD_min_ | 4 | 8.7 ± 7.0 | 0 (9) | 0.52 | 3.22 | 6.66 | 11.49 | 22.43 |
|  | PD_max_ | 4 | 10.9 ± 8.6 | 0 (9) | 0.60 | 3.32 | 8.10 | 14.98 | 25.55 |
| SET-III | HD_min_ | 12 | 33.7 ± 35.3 | 0 (4) | 2.60 | 10.14 | 22.11 | 38.65 | 119.26 |
|  | HD_max_ | 9 | 32.1 ± 23.8 | 0 (4) | 2.70 | 13.18 | 29.92 | 44.77 | 78.07 |
|  | WD_min_ | 3 | 6.8 ± 6.4 | 0 (4) | 0.41 | 2.44 | 5.00 | 8.79 | 19.87 |
|  | WD_max_ | 3 | 9.9 ± 8.0 | 0 (4) | 0.65 | 3.60 | 8.23 | 14.21 | 26.72 |
|  | PD_min_ | 4 | 13.8 ± 9.2 | 0 (4) | 2.13 | 5.90 | 12.33 | 20.23 | 31.53 |
|  | PD_max_ | 4 | 8.3 ± 6.2 | 0 (4) | 0.52 | 3.77 | 7.52 | 13.75 | 19.07 |
