# Supplementary figures and images for: Gait kinematics at trot before and after repeated ridden exercise tests in young Friesian stallions during a fatiguing 10-week training program
Source: Front Vet Sci. 2025 Feb 10;12:1456424. doi: 10.3389/fvets.2025.1456424 (PMC11848856; doi:10.3389/fvets.2025.1456424)

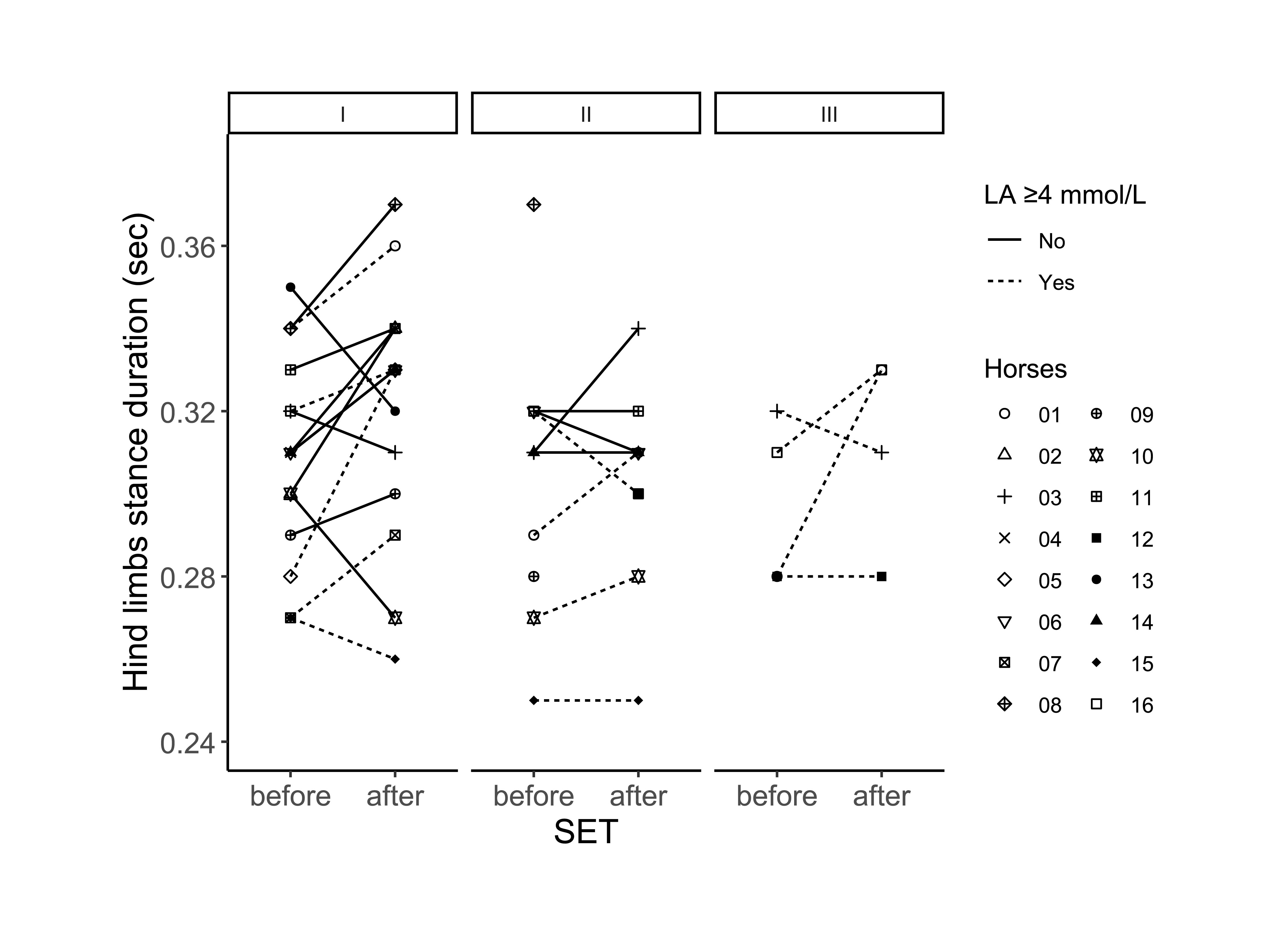

Supplement: Supplementary file 2 [file Image_1.jpg]

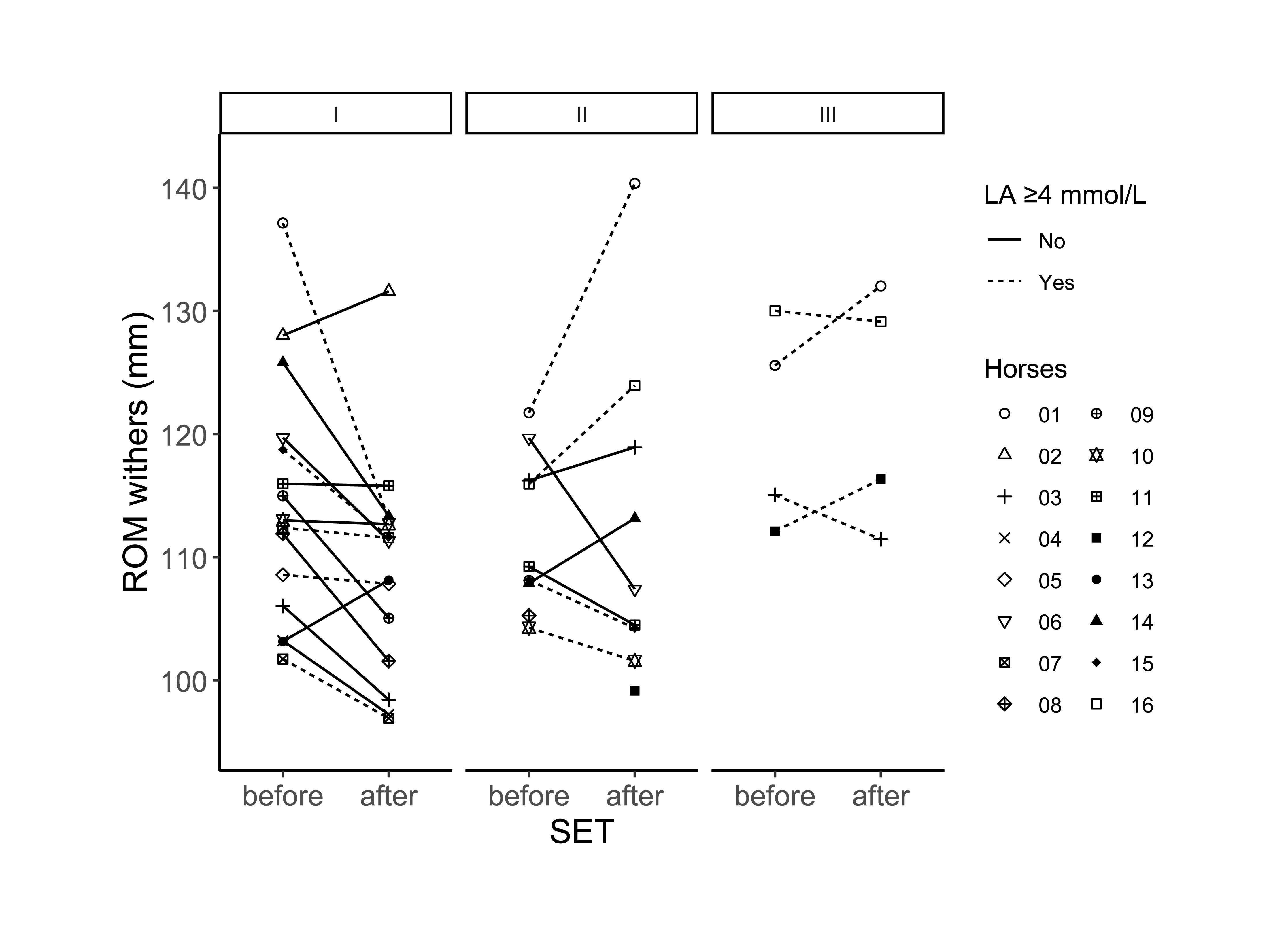

Supplement: Supplementary file 3 [file Image_2.jpg]

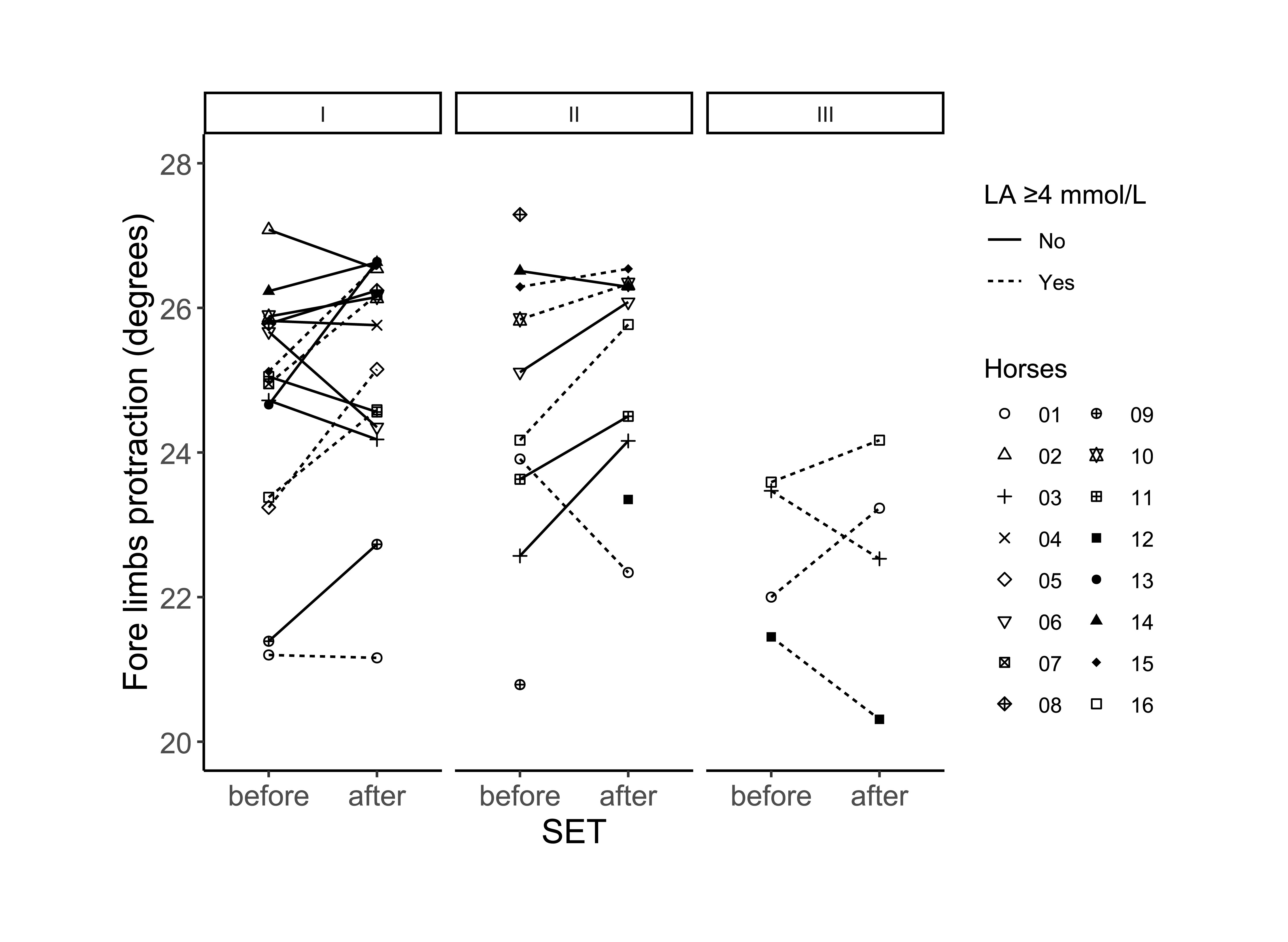

Supplement: Supplementary file 4 [file Image_3.jpg]
